# The Antiviral Factor SERINC5 Impairs the Expression of Non-Self-DNA

**DOI:** 10.3390/v15091961

**Published:** 2023-09-20

**Authors:** Yuhang Shi, Sydney Simpson, Shahad K. Ahmed, Yuexuan Chen, Aidin Tavakoli-Tameh, Sanath Kumar Janaka, David T. Evans, Ruth Serra-Moreno

**Affiliations:** 1 Microbiology and Immunology, University of Rochester Medical Center, Rochester, NY 14620, USA; yuhang_shi@urmc.rochester.edu (Y.S.); shahadk_ahmed@urmc.rochester.edu (S.K.A.); yuexuan_chen@urmc.rochester.edu (Y.C.); 2 QuidelOrtho, Rochester, NY 14626, USA; 3 Pharmaceutical Product Development, Middleton, WI 53562, USA; aidin.tavakoli-tameh@ppd.com; 4 Kelonia Therapeutics, Boston, MA 02210, USA; sjanaka@keloniatx.com; 5 Pathology and Laboratory Medicine, University of Wisconsin-Madison, Madison, WI 53792, USA; dtevans2@wisc.edu; 6 Wisconsin National Primate Research Center, Madison, WI 53715, USA

**Keywords:** SERINC5, HIV, ectopic DNA, non-self-DNA, transcriptional restriction

## Abstract

SERINC5 is a restriction factor that becomes incorporated into nascent retroviral particles, impairing their ability to infect target cells. In turn, retroviruses have evolved countermeasures against SERINC5. For instance, the primate lentiviruses (HIV and SIV) use Nef, Moloney Murine Leukemia Virus (MLV) uses GlycoGag, and Equine Infectious Anemia Virus (EIAV) uses S2 to remove SERINC5 from the plasma membrane, preventing its incorporation into progeny virions. Recent studies have shown that SERINC5 also restricts other viruses, such as Hepatitis B Virus (HBV) and Classical Swine Fever Virus (CSFV), although through a different mechanism, suggesting that SERINC5 can interfere with multiple stages of the virus life cycle. To investigate whether SERINC5 can also impact other steps of the replication cycle of HIV, the effects of SERINC5 on viral transcripts, proteins, and virus progeny size were studied. Here, we report that SERINC5 causes significant defects in HIV gene expression, which impacts virion production. While the underlying mechanism is still unknown, we found that the restriction occurs at the transcriptional level and similarly affects plasmid and non-integrated proviral DNA (ectopic or non-self-DNA). However, SERINC5 causes no defects in the expression of viral RNA, host genes, or proviral DNA that is integrated in the cellular genome. Hence, our findings reveal that SERINC5’s actions in host defense extend beyond blocking virus entry.

## 1. Introduction

Host–virus interactions have been extensively studied with the goal of uncovering novel information that would help fight virus infections. In this regard, the study of restriction factors—host proteins that inhibit virus replication at specific steps of the virus life cycle—has gained momentum in recent years, since enhancing their activity represents a promising approach against virus pathogens. To reach this goal, we need to understand how viruses interact (and evolve) with such restriction factors. One of these molecules is SERINC5. SERINC5 was identified in 2015 independently by two groups as a restriction factor that blocks HIV entry and is counteracted by the virus protein Nef [1,2].

SERINC proteins, part of a family of multi-pass transmembrane proteins, are highly conserved among eukaryotes. However, the cellular and physiological functions of SERINCs are largely unknown. The consensus is that they participate in the incorporation of the serine amino acid into lipid membranes and that they are involved in the biosynthesis of sphingolipids and phosphatidylserine by adding serine into cellular membranes [3]. Within the members of this family, SERINC5 and, to a lesser extent, SERINC3 were identified as restriction factors that block HIV infection at an early stage of the virus life cycle [2,4]. Specifically, SERINC3/5 become incorporated into budding HIV particles. Although their incorporation does not seem to affect progeny size, they significantly impair the ability of nascent virions to successfully infect a new target cell. This is due to the fact that SERINC3/5 selectively inactivate retroviral Env by: (i) altering the conformation of Env trimers, which makes them less fusogenic and more sensitive to neutralizing antibodies, and (ii) disrupting the distribution and cluster formation of Env, which inhibits the enlargement of the viral fusion pore [2,4,5,6,7]. In addition to its impact on Env, a recent study has reported that virion-associated SERINC5 exerts a post-integration block that contributes to the infectivity defect observed on SERINC5-harboring virions [8]. Conversely, HIV has evolved the virus protein Nef to counteract SERINC5. Nef is a multifunctional accessory protein that downregulates several membrane molecules by hijacking the cellular trafficking machinery, and these actions are critical for HIV pathogenesis [9,10,11,12,13,14,15]. Using a similar mechanism, Nef prevents the incorporation of SERINC5 into nascent virions by promoting SERINC5 internalization from the plasma membrane through a Clathrin- and Adaptor Protein complex 2 (AP2)-dependent endolysosomal mechanism [1,16]. In addition to Nef, the Env protein of some HIV-1 strains has been reported to overcome restriction by SERINC5 without excluding it from viral particles. In this case, it seems that certain Env trimers are inherently more fusogenic, which would in turn circumvent SERINC5’s actions [5].

Besides HIV, SERINC5 is also involved in the restriction of Simian Immunodeficiency Virus (SIV) and other retroviruses, such as Moloney Murine Leukemia Virus (MLV) and Equine Infectious Anemia Virus (EIAV). Similar to HIV, these retroviruses have evolved specific countermeasures against SERINC5, namely Nef, GlycoGag, and S2, respectively [17,18]. Recent studies have uncovered that SERINC5 can similarly affect virus entry for coronaviruses and influenza viruses [19,20]. Of note, SERINC5 has also been reported to inhibit other enveloped viruses, including Hepatitis B Virus (HBV) and Classical Swine Fever Virus (CSFV), although this restriction does not affect virus entry. In the case of HBV, SERINC5 interferes with the glycosylation of the HBV envelope glycoproteins, thereby suppressing virion production [21]. In the case of CSFV, SERINC5 inhibits virus replication by enhancing MDA5-mediated type I interferon signaling, an important innate immune response against RNA viruses [22]. These findings indicate that SERINC5 is a potent antiviral factor against many viruses. Therefore, a better understanding of the extent of the antiviral functions of SERINC5 is critical for the design of SERINC5-based therapeutic approaches aimed at treating virus infections.

Here, we uncovered a previously unappreciated role of SERINC5 in the restriction of non-self or ectopic DNA by limiting its transcription. While SERINC5 affects mRNA synthesis of lentiviral and plasmid DNA, it does not affect cellular genes nor the synthesis of foreign RNAs, like that of an RNA virus. Similar to its effect on virus entry, this activity of SERINC5 is counteracted by HIV and SIV Nef. Overall, these observations indicate that the roles of SERINC5 in host defense extend beyond viruses and that it can also protect from multiple DNA pathogens.

## 2. Materials and Methods

### 2.1. Cells and Culture Conditions

HEK293T and VeroE6 cells were purchased from ATCC (#CRL-11268 and #CRL-1586, respectively) and cultured in complete medium (Dulbecco’s Modified Eagle Medium (DMEM, ThermoFisher Scientific, Waltham, MA, USA #11885-084), supplemented with 10% Fetal Bovine Serum (FBS, ThermoFisher Scientific, #26140-079), 1% Penicillin–Streptomycin (ThermoFisher Scientific, #15070-063), and 1% L-glutamine (ThermoFisher Scientific, #25030-081)). JurkatTAg SERINC5 knockout cells were provided by Dr. Heinrich Gottlinger (University of Massachusetts Medical School). Parental JurkatTAg cells were engineered in the Evans’ laboratory to harbor an HA tag on the fourth extracellular loop of the endogenous SERINC5 protein. Cells were serially diluted after CRISPR editing for clonal selection and expansion, and the presence of the HA tag on both *SERINC5* chromosome copies was verified. These cells were cultured in Roswell Park Memorial Institute (RPMI) 1640 Medium (ThermoFisher Scientific, #11875-093), supplemented with 10% FBS, 1% Penicillin–Streptomycin, 1% L-glutamine, and 25 nM HEPES (ThermoFisher Scientific, #15630-080). HEK293T cells stably expressing GFP and HEK293T-ACE2 cells stably expressing empty vector (pQCXIP) or SERINC1 were grown in complete medium with 1 μg/mL of puromycin (ThermoFisher Scientific, #A1113803). HEK293T-ACE2-SERINC5 stable cells were cultured in complete medium with 100 μg/mL of Hygromycin B (Sigma-Aldrich, St. Louis, MO, USA #H0654).

### 2.2. Plasmid Constructs

*SERINC constructs*. The plasmid encoding human *SERINC1* (pCMV6-SERINC1-Myc-Flag) harbors a Myc-Flag tag in its C-terminus and was purchased from OriGene, Rockville, MD, USA (#RC206001). *SERINC1* was subcloned from that plasmid into the retroviral vector pQCXIP (Clontech, Mountain View, CA, USA #631516) for the generation of HEK293T-ACE2 cells stably expressing SERINC1. The retroviral vector encoding human *SERINC5* (pQCXIH-SERINC5-HA) harbors an HA tag in its fourth extracellular loop and was engineered in the Evans’ lab (University of Wisconsin-Madison). 

*GFP construct*. The pCGCG expression vector harboring *EGFP* was a gift from Dr. Jacek Skowronski, Case Western Reserve University, Cleveland, OH.

*Lentiviral constructs*. HIV-1 pNL4-3 (#ARP-114) and NL4-3Δ*nef* (#ARP-12755) were obtained through the NIH HIV Reagent Program, Division of AIDS, NIAID, NIH, Manassas, VA, USA from Dr. Malcolm Martin and Dr. Olivier Schwartz, respectively. SIV_mac_239 and its *nef*-deleted mutant were a gift from Dr. Ronald C. Desrosiers, University of Miami. The lentiviral vector harboring *GFP*, pGFP-C-Lenti, was purchased from OriGene (#TR30021). 

*Plasmids for the generation of viral-like particles* (VLPs). To generate retroviral-like particles, the Addgene plasmid #14887 (Watertown, MA, USA), which encodes for the Moloney Murine Leukemia Virus (MLV) Gag-Pol polyprotein, was used. The plasmid was deposited by Dr. Tannishtha Reya [23]. To generate lentiviral-like particles, the lentiviral packaging construct psPAX2 (NIH HIV Reagent Program, #ARP-11348) was used. The expression vector coding for the envelope glycoprotein of VSV, pMD2-G (Addgene plasmid #12259), was deposited by Dr. Didier Trono.

*Transient transfection assays*. Here, 10^6^ HEK293T cells were seeded in 6-well plates. 24 h later, cells were co-transfected with 1500 ng of proviral DNA plasmids (SIV or HIV) and 500 ng of plasmids coding for *SERINC1/5*. In the case of titration assays, cells were transfected with 1500 ng of proviral DNA and 63–1000 ng of SERINC constructs. Differences in the total DNA amount were offset by adding an empty vector.

### 2.3. Lentiviruses, Retroviral-like Particles, and Lentiviral-like Particle Preparation

*Lentiviruses and VLPs*. Lentiviruses and virus-like particles were propagated at the Serra-Moreno biosafety level 2^+^ (BSL2^+^) lab following the approved standard operating procedures.

*Lentivirus stock production*. Here, 5 × 10^6^ HEK293T cells were seeded in 10 cm^2^ dishes. 24 h later, cells were transfected with 5 μg of HIV-1 pNL4-3 or NL4-3Δ*nef*. 48 h later, the supernatants were collected, and debris was removed by centrifugation for 10 min at 931× *g*. Next, the supernatants were aliquoted in 1 mL cryovials and stored at −80 °C.

*Production of VLPs harboring genes of interest* (pQCXIP, *GFP*, *SERINC1*, and *SERINC5*). In order to transduce cells with genes of interest, retroviral- or lentiviral-like particles were produced. For this, 5 × 10^6^ HEK293T cells were seeded in 10 cm^2^ dishes. 24 h later, cells were transfected with 3.75 μg of a packaging plasmid (either MLV Gag-Pol for retroviral-like particles or psPAX2 for lentiviral-like particles), 1.25 μg of the VSV-G-expressing plasmid, and 5 μg of a retroviral or lentiviral plasmid encoding the gene of interest. 48 h later, the supernatant was collected, debris was removed by centrifugation for 10 min at 931× *g*, and the supernatant was aliquoted in 1 mL cryovials, which were later stored at −80 °C.

### 2.4. Virus Infection and Transduction Assays

*HIV*. All HIV infections and VLP transduction experiments were performed at the Serra-Moreno BSL2^+^ lab, following the approved standard operating procedures. 100 ng of p24 equivalents of HIV-1 NL4-3 or NL4-3Δ*nef* were spinoculated into 10^6^ JurkatTAg SERINC5-KO or SERINC5 HA cells at 1650× *g* at 4 °C for 1 h to synchronize the infections. Cells were then washed, resuspended in complete medium, seeded in 24-well plates, and incubated at 37 °C. 16 h post-infection, the supernatants were collected to assess virion production by ELISA, and cells were harvested for western blot and RT-qPCR analyses. 

*Transduction*. To generate cells stably expressing genes of interest, 3 × 10^6^ HEK293T or HEK293T-ACE2 cells were seeded in T25 flasks. 24 h later, cells were infected with 1 mL of stock pQCXIP, GFP, SERINC1, or SERINC5 VLPs for 2 h at 37 °C. One day post-transduction, cells were cultured in complete medium with 1 μg/mL of puromycin or 100 μg/mL of Hygromycin B. The stable cell lines were verified after 2 weeks of antibiotic selection by western blot and flow cytometry.

*SARS-CoV-2*. All SARS-CoV-2 infection experiments were performed at the URMC BSL3 laboratory following the approved standard operating procedures. Here, 10^6^ HEK293T-ACE2 cells stably expressing an empty vector (pQCXIP), SERINC1, or SERINC5 were seeded in 6-well plates. 24 h later, cells were infected with SARS-CoV-2 Hong Kong (BEI, #NR-52282, Manassas, VA, USA) at MOI (Multiplicity Of Infection) of 1. As controls, we included non-treated cells (NT) and mock-infected cells, which consisted of VLPs harboring *GFP*. One hour post-infection, cells were washed, and fresh medium was added. Then, 8, 24, and 48 h post-infection, supernatants were collected to assess infectious particle production, and cells were harvested for western blot and RT-qPCR analyses. 

### 2.5. ELISA

HIV-1 NL4-3 and NL4-3 Δ*nef* virus stocks and culture supernatants, obtained from either HEK293T cells or through infectivity assays in JurkatTAg cells, were titered by ELISA (HIV-1 p24 antigen-capture assay, ABL inc., Rockville, MD, USA, #5421), following the manufacturer’s instructions. Similarly, SIV_mac_239 and SIVΔ*nef* culture supernatants obtained from HEK293T cells were titered by ELISA (SIV p27 antigen-capture assay, ABL inc., #5436), following the manufacturer’s instructions. Differences in virus particle release between SERINC5^+^ and SERINC5^−^ cells were expressed as the percentage of virion production, in which particle production from SERINC5^−^ cells was considered as the maximum release (100%).

### 2.6. Median Tissue Culture Infectious Dose Assay (TCID_50_)

For this, 2.5 × 10^4^ VeroE6 cells were seeded in 96-well plates. The supernatants recovered from SARS-CoV-2 infections were serially diluted (10^−1^ to 10^−9^) in DMEM with 3% FBS. Cell media were removed, and cells were infected with 100 μL of virus dilutions in six replicates. Infected cells were incubated at 37 °C. Three days post-infection, the cytopathic effect (CPE) on each well was determined by optical microscopy. The TCID_50_ of viruses was calculated using the Spearman–Kärber method [24]. 

### 2.7. Western Blot

Cells subjected to western blot analyses were washed with DPBS (ThermoFisher Scientific, #14190-144) and harvested by adding lysis IP buffer (ThermoFisher Scientific, #87787). Cells were then kept on ice for 30 min. Cell lysate was cleared by centrifugation at 16,000× *g* at 4 °C for 8 min. Next, the supernatants were mixed with 2× SDS sample buffer (Sigma-Aldrich, #S3401), and samples were boiled for 10 min on a heat block. In case of SARS-CoV-2-infected cells, lysis buffer was supplemented with 1% Triton X-100 (Sigma-Aldrich, #X100). Proteins were separated using 12% SDS-PAGE polyacrylamide gels. Proteins were then transferred to a polyvinylidene difluoride (PVDF) membrane (Bio-Rad, Hercules, CA, USA #1620264) using a Trans-Blot Turbo Transfer System (Bio-Rad). Membranes were incubated for 1 h with 5% non-fat milk (Bio-Rad, #1706404XTU) at room temperature, followed by an overnight incubation with the primary antibodies (Table 1) at 4 °C. Next, membranes were washed 3 times with PBS-tween (Sigma-Aldrich, #P3563) followed by 1 h incubation with the secondary antibodies (Table 1) at room temperature. Subsequently, three additional washes with PBS-tween were performed before imaging the membranes. Finally, membranes were developed by adding the SuperSignal West Femto maximum-sensitivity substrate (ThermoFisher Scientific, #34095), and proteins were visualized in a ChemiDoc imaging system (Bio-Rad). The expression level of proteins was quantified using ChemiDoc Image Lab software, version 6.1 (Bio-Rad). Each experiment was repeated three independent times.

### 2.8. Fluorescence Microscopy

To visualize SERINC5 and SERINC1, 6 × 10^5^ HeLa cells were seeded in 6-well plates. 24 h later, cells were co-transfected with 1000 ng of SERINC1-Myc-Flag and SERINC5-HA constructs. The following day, 3 × 10^4^ cells were plated onto tissue culture-treated 8-well microscopy slides. 48 h post-transfection, cells were washed with ice-cold DPBS three times. Next, cells were fixed and permeabilized by adding 50:50 acetone:methanol (Sigma-Aldrich, #270725 and #34860) for 10 min at −20 °C. Cells were then blocked with antibody diluent solution (2% fish skin gelatin (Sigma-Aldrich) + 0.1% Triton X-100 (Sigma-Aldrich) + 10% goat serum (ThermoFisher Scientific, #67765) in 1 × DPBS) for 30 min at room temperature, followed by an incubation of 1 h with a primary antibody cocktail at room temperature (anti-Myc-tag rabbit polyclonal at 1:200 and an anti-HA-tag mouse IgG_1_ at 1:200, see Table 1). Next, cells were washed with wash buffer (2% fish skin gelatin + 0.1% Triton X-100 in 1 × DPBS) three times and incubated with a secondary antibody cocktail (Alexa-488 anti-mouse IgG_1_ and Alexa-568 anti-rabbit IgG, both at 1:500; Table 1) for 30 min. After washing 3 times, cells were incubated with DAPI at a 1:5000 dilution (Table 1) for 5 min to visualize the nuclei. Finally, the slides were washed and mounted using anti-quenching mounting medium (Vector Laboratories, Newark, CA, USA #3304770) and they were visualized using a BioTek Lionheart FX automated microscope using 20× and 40× objectives and filter cubes of 377, 469, and 586 nm. Images were processed and analyzed using the Gen5 software version 3.14 (BioTek Instruments, Winooski, VT, USA). 

### 2.9. RT-qPCR Assays

*RNA extraction and cDNA synthesis*. Cells with different treatments (transfection, infection, drug treatments) were harvested, washed with DPBS, and total RNA was extracted using the Qiagen RNeasy Mini-kit (Germantown, MD, USA #74004), following the manufacturer’s instructions. In the case of cells infected with SARS-CoV-2, total RNA was extracted by adding 1 mL of Trizol (ThermoFisher Scientific, #15596018) per well. Then, 200 μL of chloroform (Spectrum Chemical, New Brunswick, NJ, USA #C1220) was added, and samples were centrifuged at 12,000× *g* for 15 min at 4 °C to create three phases of separation: a lower red phenol-chloroform phase, an interphase, and a transparent upper aqueous phase. The aqueous phase was collected and mixed with 500 μL of isopropyl alcohol (Sigma-Aldrich, #W292907) to precipitate RNA. RNA was then washed with 75% ethanol and eluted in RNase-free water (ThermoFisher Scientific, #10977-025). The concentration and A260/A280 ratio of RNA were measured by a NanoDrop (ThermoFisher Scientific). Next, 1 μg of purified RNA was converted into cDNA using the iScript cDNA synthesis kit (Bio-Rad, #1725037), following the manufacturer’s instructions.

*qPCR*. The mRNA levels of genes of interest were measured by the SYBR green-based real-time qPCR method. For each sample, different controls, including RNA quality (RQ1 and RQ2), genomic DNA contamination (gDNA), and housekeeping genes (*GAPDH*, *Calreticulin*), were measured by qPCR. In each PCR reaction, 10 μL of 2× SsoAdvanced Universal SYBR Green Supermix (Bio-Rad, #1725272), 0.2 μL of cDNA, 8.8 μL of RNase-free water (ThermoFisher Scientific, #10977-015), and 1 μL of the primer pair for the target gene or control were included. The amplification program was as follows: 2 min at 95 °C for initial activation, 40 cycles at 95 °C for 5 s, 60 °C for 30 s, and then melting analyses from 65 to 95 °C (0.5 °C increments). Each sample was analyzed by qPCR in two technical replicates on a CFX-96 BioRad instrument. Assays were performed three independent times for each experimental condition. The following qPCR primers were purchased from BioRad: Human Calreticulin (#qHsaCID0016904), Human GAPDH (#qHsaCED0038674), Human gDNA (#qHsaCtlD0001004), Human LC3B (#qHsaCED0038576), Human RQ1/RQ2 (#qHsaCtlD0001002), and Human SERINC1 (#qHsaCID0016529). Additional primers for qPCR were synthesized from Integrated DNA Technologies (Coralville, IA, USA): GFP (FW: 5′CAAACTGCCTGTTCCATGGC3′, RV: 5′CCTTCGGGCATGACACTCTT3′), SARS-CoV-2 NSP6 (FW: 5′ATGGTGCTAGGAGAGTGTGG3′, RV: 5′AGAGCCCACATGGAAATGGC3′), SARS-CoV-2 RdRp (FW: 5′AATAGAGCTCGCACCGTAGC3′, RV: 5′CATGTTGTGCCAACCACCAT3′), HIV NL4-3 Gag (FW: 5′TATCAGAAGGAGCCACCCCA3′, RV: 5′CCCATTCTGCAGCTTCCTCA3′), HIV NL4-3 Tat (FW: 5′GCGACGAAGAGCTCATCAGA3′, RV: 5′CTATTCCTTCGGGCCTGTCG3′), HIV NL4-3 splicing donor site 1 (FW: 5′CAAGAGGCGAGGGGCGGCGA3′), HIV NL4-3 splicing accepting site 1 (RV: 5′CTTGGCACTACTTTTATGT3′), HIV NL4-3 splicing accepting site 2 (RV: 5′CTAGGACTAACTATACGT3′), HIV NL4-3 splicing accepting site 3 (RV: 5′ACTTCCTGGATGCTTCCAG3′), HIV NL4-3 splicing accepting site 5 (RV: 5′ACTATTATAGGTTGCATTA3′), and Human SERINC5 (FW: 5′ATCGAGTTCTGACGCTCTGC3′, RV: 5′GCTCTTCAGTGTCCTCTCCAC3′). Differences in expression were calculated as the fold-change over SERINC1, vector, or SERINC5-KO cells. A fold-change of >2.0 or <0.5 relative to the control samples was considered biologically relevant [25].

### 2.10. DNA Isolation

Here, 5 × 10^5^ HEK293T cells were seeded in 6-well plates. 24 h later, cells were co-transfected with 1500 ng of pCGCG and 500 ng of plasmids encoding either an empty vector, *SERINC1*-Myc-Flag, or *SERINC5*-HA. Cells were harvested at 4, 24, 48, and 96 h post-transfection. DNA was isolated at each time point using a Genomic DNA Extraction Kit (Qiagen, #69504), following the manufacturer’s instructions. The concentration and A260/A280 ratios of the DNA samples were measured by a NanoDrop (ThermoFisher Scientific). Then, 50 ng of isolated DNA were amplified by qPCR to assess for the DNA levels of SERINC1/5, host input DNA, and GFP plasmids, using the primers and procedures described above.

*Integrated HIV DNA*. qPCR analyses were also performed to measure the relative levels of integrated HIV DNA. Here, 10^6^ JurkatTAg SERINC5 knockout cells, and parental JurkatTAg cells harboring an HA tag on the fourth extracellular loop of endogenous SERINC5, were spinoculated with 500 ng of p24 equivalents of NL4-3 Δ*nef* at 1650× *g* and 4 °C for 1 h to synchronize the infections. Cells were then washed, resuspended in complete medium, seeded in 24-well plates, and incubated at 37 °C. 16 h later, cells were harvested, and total DNA was extracted using a Genomic DNA Extraction Kit (Qiagen, #69504). Integrated HIV DNA was measured by Alu-qPCR through a 2-step PCR reaction. First, 50 ng of total DNA was amplified using Alu-FW (5′GCCTCCCAAAGTGCTGGGATTACAG3′) and Gag-RV (5′GTTCCTGCTATGTCACTTCC3′) primers. Next, 2 μL of the purified PCR product were amplified by qPCR using R-FW (5′TTAAGCCTCAATAAAGCTTGCC3′) and U5-RV (5′GTTCGGGCGCCACTGCTAGA3′) primers, as described previously [26]. To ensure a comparable DNA input across samples, qPCR primers against host genomic DNA and GAPDH were used (see Section 2.8). To examine whether differences in DNA integration are caused by changes in infectivity between parental and SERINC5-KO cells, additional infections were performed. Total RNA was harvested at time zero (right after spinoculation, to measure the degree of attached virions), and 2 h post-infection (to measure viral uptake). The amounts of HIV genomic RNA were measured by RT-qPCR using the NL4-3 Gag FW and RV primers described in Section 2.8. 

### 2.11. Drug Treatments

To measure the impact of SERINC5 on HIV protein degradation, the lysosomal inhibitor Hydroxychloroquine (60 μM; Sigma-Aldrich, #H0915) and the proteasomal inhibitor ALLN (25 μM; Sigma-Aldrich, #208719) were added 18 h before samples were collected. Any fluctuations in host and virus protein expression were measured by western blot.

To examine the effect of SERINC5 on RNA degradation, the transcriptional inhibitor Actinomycin D (5 μg/mL; Sigma-Aldrich, #A9415) was added 24 h post-transfection. Cells were harvested at 0, 4, 8, and 24 h after adding Actinomycin D, and the RNA levels of *GFP* were measured by RT-qPCR, as described above.

As controls for the HIV integration assays, parental and SERINC5-KO Jurkat TAg cells were treated with Raltegravir (an integrase inhibitor; HIV Reagent Program # 11680; 260 nM) and Efavirenz (a non-nucleoside reverse transcriptase inhibitor; HIV Reagent Program #4624; 40 μM).

### 2.12. Statistical Analysis

Statistical calculations were performed using the two-tailed, unpaired Student’s T test analysis. All statistical analyses were performed using GraphPad Prism version 10. *p* values ≤ 0.05 were considered statistically significant.

## 3. Results

### 3.1. SERINC5 Suppresses Lentivirus Virion Production by Downregulating the Structural Protein Gag

**Figure 1:**
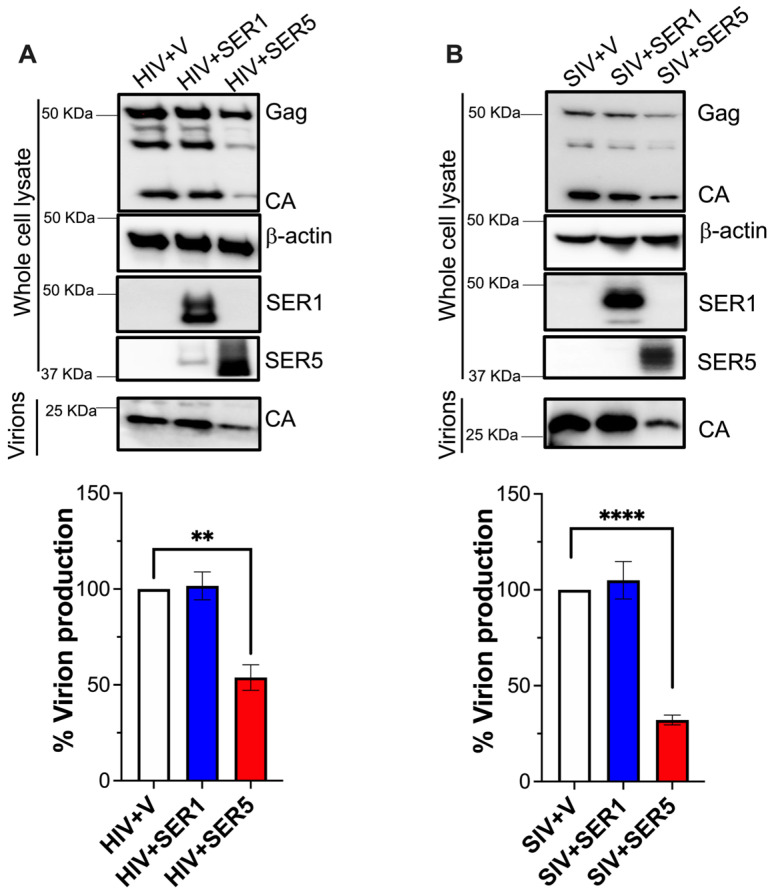
SERINC5 downregulates lentivirus Gag and consequently reduces virion production. HEK293T cells were co-transfected with HIV-1 NL4-3 (**A**) or SIV_mac_239 (**B**) proviral DNA and either an empty vector, SERINC1-Myc-Flag, or SERINC5-HA constructs. Forty-eight hours post-transfection, cells and supernatants were harvested, and protein expression was analyzed by western blot (**top** panels). The culture supernatants from these transfections were examined for virion production by p24- or p27-ELISA and expressed as the percentage of virion production (graphs). **: *p* < 0.01, ****: *p* < 0.0001. Blots are representative of 3 independent assays. Data correspond to the mean and SEM of 3 independent experiments.

It is well known that SERINC5 blocks HIV and SIV entry, and that this effect is counteracted by Nef [1,2]. More recently, SERINC5 has been reported to impact virion production and the synthesis of virus proteins for other enveloped viruses. These actions differ from the restriction in entry that SERINC5 exerts on retroviruses [8,21,22]. To examine if SERINC5 could similarly impact virion production in lentiviruses, the levels of the virus protein Gag and its mature product capsid (CA) were measured in virion-producing cells as well as in culture supernatants. We focused on Gag because it is the major driver of virion assembly and release. For this, HEK293T cells were co-transfected with HIV-1 NL4-3 or SIV_mac_239 proviral DNA, along with a construct expressing human *SERINC5*-HA. HEK293T cells were selected for these assays due to their high transfection efficiency and because they do not harbor the HIV cellular receptors, so virions generated by transfection cannot infect these cells—which allows us to exclude any role of SERINC5 in blocking entry and impacting virus protein levels over multiple rounds of replication. Constructs coding for human *SERINC1*-Myc-Flag or an empty vector were included as controls. SERINC1 is another member of the SERINC family and has a similar protein topology and localization as SERINC5 [3,27,28,29]. In fact, SERINC1 and SERINC5 subcellular distribution highly overlaps (Supplementary Figure S1). However, SERINC1 has no known antiviral effect on retroviruses [30]. Unlike cells transfected with the empty vector or SERINC1, cells expressing SERINC5 displayed lower expression levels of the HIV and SIV structural protein Gag, which consequently impacted the amounts of CA detected in the culture supernatants (Figure 1A,B, top). Consistent with this result, virion production, which was measured by CA-specific ELISA, was significantly reduced in SERINC5-expressing cells (Figure 1A,B, bottom). Hence, these findings indicate that SERINC5 causes the downregulation of lentiviral Gag and consequently reduces virion production.

### 3.2. SERINC5 Causes a Decrease in Lentivirus Proteins, but This Effect Is Counteracted by Nef

**Figure 2:**
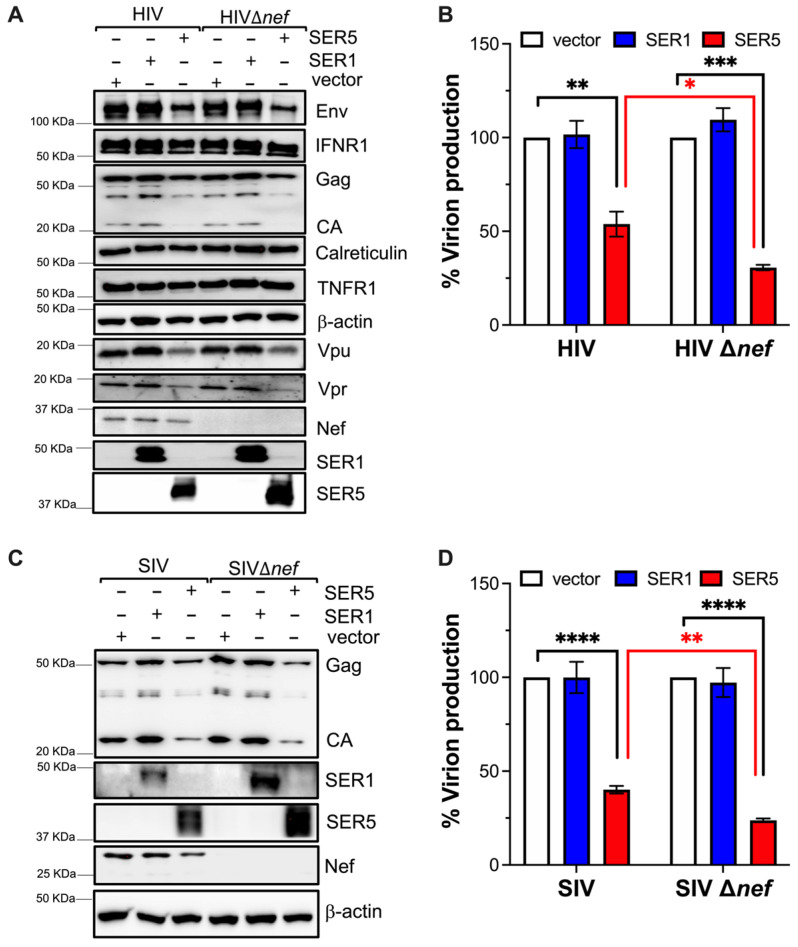
SERINC5 decreases lentivirus proteins and virion production, but this effect is counteracted by HIV and SIV Nef. (**A**,**B**) HEK293T cells were co-transfected with HIV-1 NL4-3 or NL4-3Δ*nef* and either an empty vector, SERINC1-Myc-Flag, or SERINC5-HA constructs. Forty-eight hours post-transfection, cells were harvested, and protein expression was measured by western blot (**A**). Additionally, culture supernatants were examined for virion production by p24-ELISA (**B**). (**C**,**D**) HEK293T cells were co-transfected with SIV_mac_239 or SIV_mac_239Δ*nef* and either an empty vector, SERINC1-Myc-Flag, or SERINC5-HA constructs. Forty-eight hours post-transfection, cells were harvested, and protein expression was measured by western blot (**C**). In addition, culture supernatants were examined for virion production by p27-ELISA (**D**). *: *p* < 0.05, **: *p* < 0.01, ***: *p* < 0.001, ****: *p* < 0.0001. Blots are representative of 3 independent assays. Data correspond to the mean and SEM of 3 independent experiments.

To investigate if the effect of SERINC5 on Gag is specific for this protein, new transfection experiments were performed. In addition, the proviral DNA for the *nef*-deleted NL4-3 and SIV_mac_239 viruses were included to examine if this novel activity of SERINC5 could also be counteracted by Nef. Forty-eight hours post-transfection, cells were harvested and analyzed by western blot for virus proteins as well as host proteins (to investigate if the effect of SERINC5 was virus-specific). As in Figure 1, the culture supernatants were examined for virion production by ELISA. Similar to our observations for Gag, a reduction in the HIV proteins Env, Vpu, Vpr, and Nef was detected in SERINC5-expressing cells, but not in vector- or SERINC1-expressing cells (Figure 2A and Supplementary Figure S2). In line with the results in Figure 1, a significant reduction in virion production was also observed from SERINC5-expressing cells, and this defect was more prominent for *nef*-deleted viruses (Figure 2B). Similar findings were obtained for SIV (Figure 2C,D). Of note, the level of host proteins, including those located at membranes where SERINC5 is normally found (plasma membrane: IFNR1, TNFR1, and ER: Calreticulin) remained unchanged in the presence of SERINC5 (Figure 2A). Therefore, the SERINC5-mediated downregulation of virus proteins appears to be specific. Remarkably, these additional actions of SERINC5 are also counteracted by Nef, since: (i) the expression levels of SERINC5 were decreased in the presence of wildtype (Nef-expressing) HIV and SIV, (ii) the degree of virus protein depletion was more dramatic in the absence of Nef (Figure 2A,C and Supplementary Figure S2), and (iii) virion release was less impacted in the presence of Nef (Figure 2B,D).

**Figure 3:**
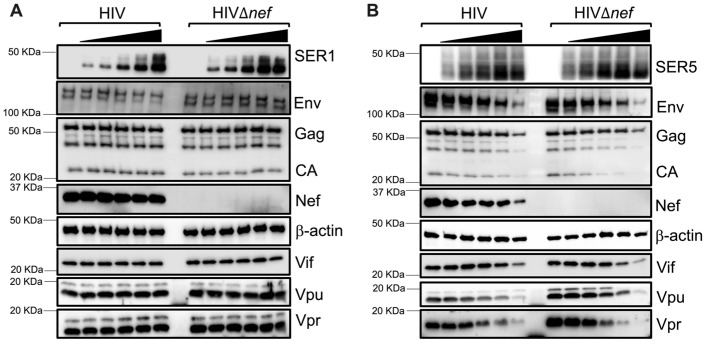
SERINC5 downregulates HIV proteins in a dose-dependent manner. HEK293T cells were co-transfected with 1500 ng of HIV-1 NL4-3 or NL4-3Δ*nef* proviral DNA and increasing amounts (63–1000 ng) of SERINC1-Myc-Flag (**A**) or SERINC5-HA (**B**). Differences in plasmid concentrations were offset by adding an empty vector. Forty-eight hours post-transfection, cells were harvested and analyzed for virus proteins by western blot. Blots are representative of 3 independent assays.

To rule out the possibility that these antiviral actions are due to an artifact of overexpressing SERINC5, a dose-dependent assay was performed. HEK293T cells were co-transfected with 1500 ng of HIV-1 NL4-3 or NL4-3Δ*nef* proviral DNA and increasing concentrations (63–1000 ng) of plasmids coding for *SERINC1*-Myc-Flag or *SERINC5*-HA. Differences in plasmid levels were offset by adding an empty vector. No impact on the HIV proteins Env, Gag, CA, Nef, Vif, Vpu, nor Vpr was detected in the presence of SERINC1 (Figure 3A). In contrast, a dose-dependent reduction in these proteins was observed in cells expressing SERINC5 (Figure 3B). Remarkably, the SERINC5 effect was noticeable even in cells transfected with the lower concentrations of the SERINC5 plasmid (Figure 3B, see lanes 2 and 3 of HIVΔ*nef* blots: Env, CA, Vpr) and was partially counteracted in the presence of Nef, supporting the notion that the phenotype observed in SERINC5-expressing cells is specific and not due to plasmid competition. This possibility was further excluded by transfecting cells with the proviral DNA and SERINC constructs on separate days. 

**Figure 4:**
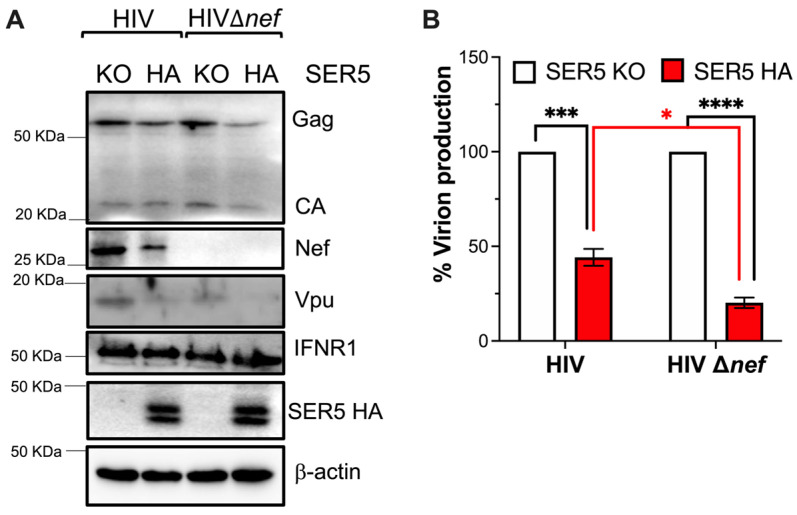
SERINC5 restricts HIV gene expression in CD4^+^ T cells. JurkatTAg SERINC5-KO and parental cells harboring an HA tag in the endogenous *SERINC5* gene were infected with 100 ng of p24 equivalents of HIV-1 NL4-3 or NL4-3Δ*nef* viruses. Sixteen hours post-infection, cells were harvested, and protein levels were measured by western blot (**A**). In parallel, the culture supernatants were examined for virion production by p24-ELISA (**B**). *: *p* < 0.05, ***: *p* < 0.001, ****: *p* < 0.0001. Blots are representative of 3 independent assays. Data correspond to the mean and SEM of 3 independent experiments.

To determine if this effect of SERINC5 is exclusive to HEK293T cells or can also be observed in the context of infection, JurkatTAg cells were used. JurkatTAg SERINC5 knockout (SER5 KO) cells, and parental JurkatTAg cells engineered to harbor an HA tag on the fourth extracellular loop of the endogenous SERINC5 protein (SER5 HA), were infected with 100 ng of CA equivalents of HIV-1 NL4-3 or NL4-3Δ*nef*. The impact of SERINC5 on virus proteins was examined 16 h post-infection—which would correspond to one full cycle of replication. This early time of analysis helped us exclude the effect of SERINC5 on blocking re-infection, which could lead to a reduction of virus proteins over multiple rounds of virus replication. The presence of SERINC5 caused a decrease in virus proteins, including Gag, Nef, and Vpu, and thus, reduced virion production (Figure 4A,B). However, no fluctuations were observed in the expression levels of host proteins (IFNR1 and β-actin) (Figure 4A). Similar to our findings in HEK293T cells, the impact of SERINC5 was more striking in the absence of HIV Nef (Figure 4A,B). These observations further support that SERINC5 causes a defect in HIV proteins, which subsequently affects virion production, but this restriction is counteracted by Nef.

### 3.3. SERINC5 Downregulates HIV Gene Expression at the mRNA Level

**Figure 5:**
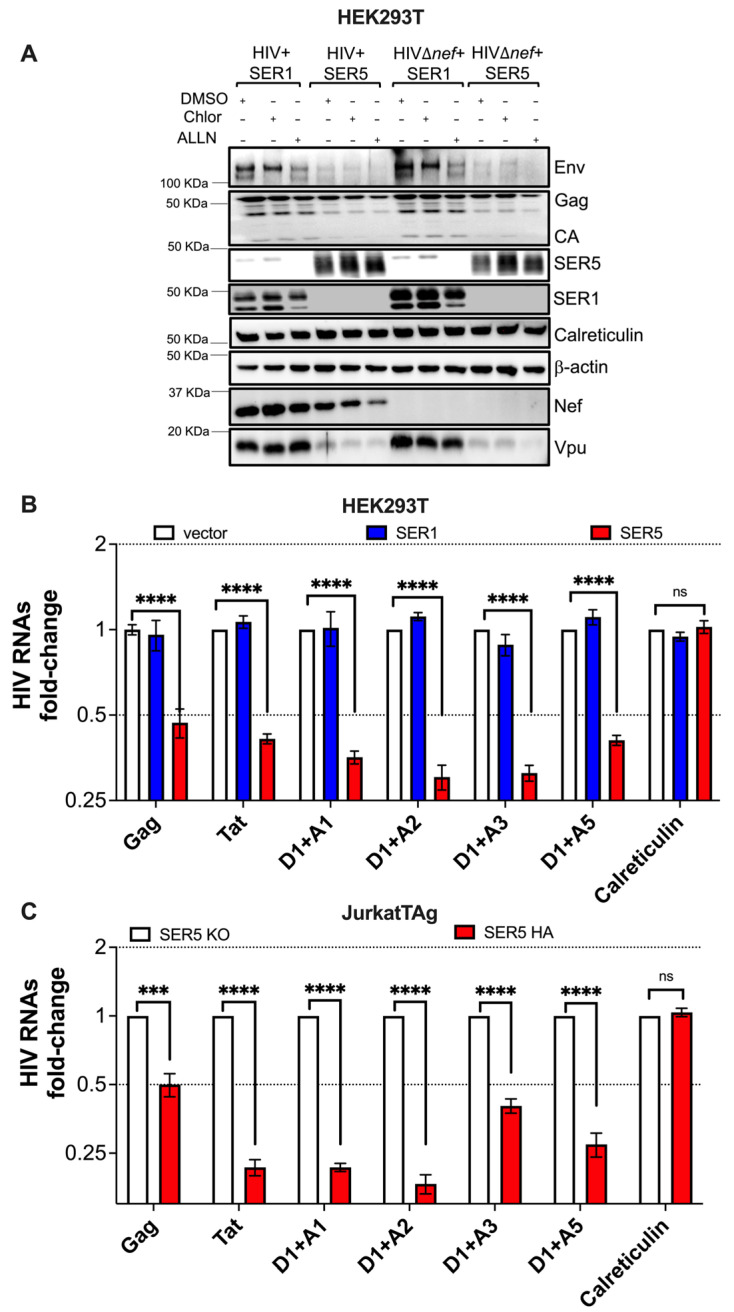
SERINC5 decreases HIV gene expression at the mRNA level. (**A**) HEK293T cells were co-transfected with HIV-1 NL4-3 or NL4-3Δ*nef* proviral DNA and either SERINC1-Myc-Flag or SERINC5-HA. Thirty hours post-transfection, DMSO, Hydroxychloroquine (Chlor), or ALLN were added. Eighteen hours later, fluctuations in HIV proteins were measured by western blot. (**B**) HEK293T cells were co-transfected with HIV-1 NL4-3Δ*nef* proviral DNA and either an empty vector, SERINC1-Myc-Flag, or SERINC5-HA constructs. Forty-eight hours later, changes in different HIV mRNA splicing variants were measured by RT-qPCR and expressed as fold-change. (**C**) Similar assays were performed in the context of infection with NL4-3Δ*nef* in JurkatTAg SERINC5-KO and parental JurkatTAg cells harboring an HA tag on the endogenous *SERINC5* gene. HIV-1 RNAs were measured 16 h post-infection. ***: *p* < 0.001, ****: *p* < 0.0001, ns: not significant. Dotted lines represent the threshold for biological significance. Blots are representative of 3 independent assays. Data correspond to the mean and SEM of 3 independent experiments.

Since the presence of SERINC5 causes defects in lentivirus protein expression, we examined whether SERINC5 drives the degradation of these proteins. To test this hypothesis, fluctuations in HIV protein levels were evaluated in the presence and absence of lysosomal and proteasomal inhibitors. HEK293T cells were co-transfected with HIV-1 NL4-3 or NL4-3Δ*nef* proviral DNA and either SERINC1-Myc-Flag or SERINC5-HA. Thirty hours later, Hydroxychloroquine (Chlor; a lysosomal inhibitor), ALLN (a proteasomal inhibitor), or DMSO (a mock control) were added. Cells were harvested 18 h later. An enhancement in SERINC5 levels was detected in cells treated with both Hydroxychloroquine and ALLN, suggesting that SERINC5 is degraded in both lysosomes and proteasomes (Figure 5A). This notion was confirmed in JurkatTAg cells, which express SERINC5 endogenously (Supplementary Figure S3). However, treatment with these inhibitors did not restore HIV protein levels (Gag, Env, Vpu, and Nef); if anything, they were further decreased, especially in the presence of the proteasomal inhibitor, which magnifies SERINC5 expression (Figure 5A, see the HIVΔ*nef* panel). These data indicate that the effect of SERINC5 on HIV proteins is not caused by promoting their degradation.

We then tested the alternative hypothesis that SERINC5 acts at the RNA level, either by affecting transcription or RNA stability. To examine these possibilities, total RNA was isolated from HEK293T cells transfected with NL4-3Δ*nef* proviral DNA and JurkatTAg cells infected with NL4-3Δ*nef* virus. Here, we used NL4-3Δ*nef* because this clone is more susceptible to SERINC5′s actions. Next, the RNA levels of the full-length HIV genomic RNA (gRNA) and other splicing species were measured by Reverse Transcription, followed by qPCR (RT-qPCR; see Supplementary Figure S4 for a schematic of the splicing variants and primers used). Whereas SERINC1 had no impact on HIV transcripts (Figure 5B), SERINC5 led to a drastic decrease in all HIV mRNAs, including full-length un-spliced (Gag), as well as single-spliced (D1 + A1, D1 + A2 and D1 + A5) and multiple-spliced (Tat and D1 + A3) species. This effect of SERINC5 on overall HIV mRNAs explains why it causes a general downregulation in HIV proteins. Importantly, mRNA levels for cellular genes (i.e., *Calreticulin*) were not affected by SERINC5 (Figure 5B,C). Taken together, these results indicate that SERINC5 restricts HIV gene expression at the mRNA level.

**Figure 6:**
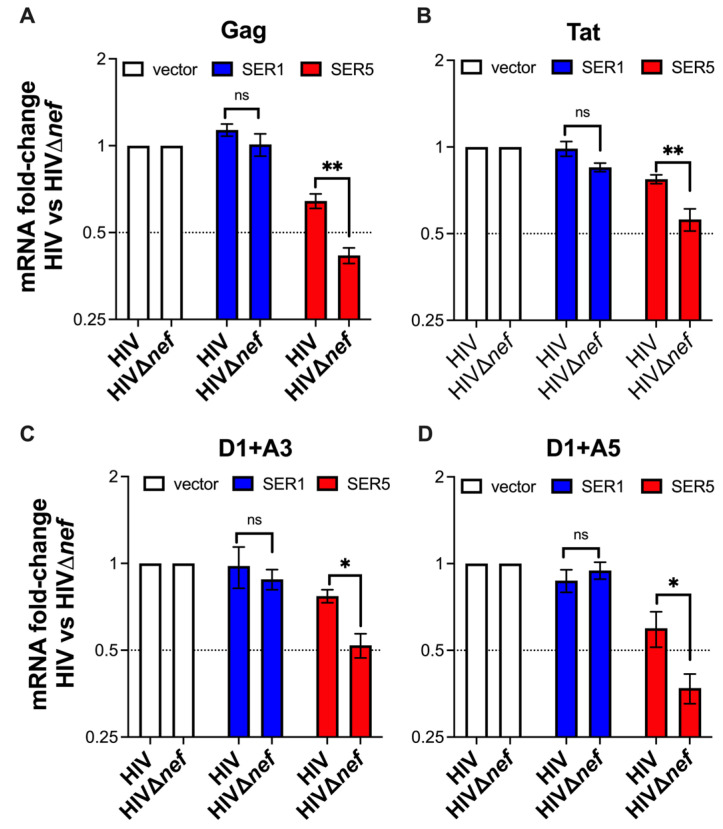
HIV Nef counteracts the SERINC5-mediated downregulation of viral RNAs. (**A**–**D**) HEK293T cells were co-transfected with HIV-1 NL4-3 or NL4-3Δ*nef* proviral DNA and either an empty vector, SERINC1-Myc-Flag, or SERINC5-HA. The expression levels of different HIV transcript species (**A**: un-spliced, **B**,**C**: multiple-spliced, **D**: single-spliced) were compared between wildtype and *nef*-deleted HIV 48 h post-transfection by RT-qPCR. *: *p* < 0.05, **: *p* < 0.01, ns: not significant. Dotted lines represent the threshold for biological significance. Data correspond to the mean and SEM of 3 independent experiments.

To corroborate that the SERINC5-mediated downregulation of HIV transcripts can be counteracted by Nef, we compared HIV mRNA yields between *nef*-competent and *nef*-defective NL4-3. While no differences in HIV RNA counts were observed between wildtype and NL4-3Δ*nef* in cells expressing SERINC1, HIV transcripts were significantly downregulated, particularly for NL4-3Δ*nef*, in SERINC5^+^ cells (Figure 6), indicating that a functional Nef can counteract this restriction.

### 3.4. SERINC5 Downregulates the Expression of Ectopic, Non-Self-DNA

**Figure 7:**
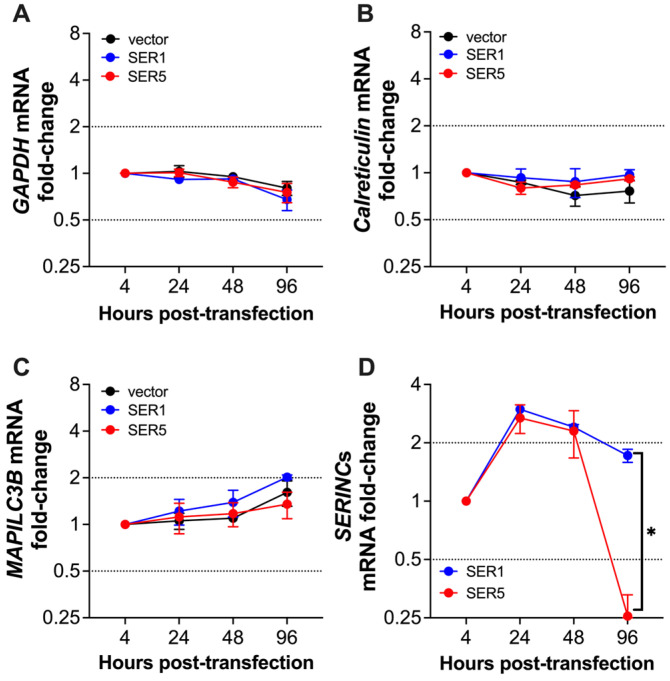
SERINC5 has no impact on the expression of cellular genes. The mRNA levels of host genes: *GAPDH* (**A**)*, Calreticulin* (**B**), and *MAPILC3B* (**C**), were measured in HEK293T cells transfected with an empty vector, SERINC1-Myc-Flag, or SERINC5-HA constructs. Changes in these cellular mRNAs were evaluated over a 4-day period by RT-qPCR. (**D**) Similarly, changes in the expression of the SERINC1-Myc-Flag and SERINC5-HA constructs were monitored by RT-qPCR during this 4-day period. *: *p* < 0.05. Dotted lines represent the threshold for biological significance. Data correspond to the mean and SEM of 3 independent experiments.

Although we did not observe a SERINC5-mediated reduction in host gene expression (Figure 2A, Figure 4A and Figure 5), we cannot completely exclude this possibility just yet. Cells already harbor mRNAs coding for their gene products, and these RNAs can have different half-lives. For instance, highly stable mRNAs can be translated before and after transfection with SERINC5, so if SERINC5 affects cellular RNAs it may take longer to see any defects in their levels than for genes that are ectopically expressed from a virus or a plasmid. To better assess whether SERINC5 affects host gene expression, we performed a time course experiment, where the mRNAs of several host genes were monitored, specifically, *GAPDH* (a gene involved in glycolysis and commonly used as a housekeeping gene [31,32]), *Calreticulin* (coding for an ER protein [33,34]), and *MAPILC3B* (also known as LC3B, coding for an autophagy-related molecule important for cell homeostasis [35,36]). HEK293T cells were transfected with SERINC1-Myc-Flag, SERINC5-HA, or an empty vector, and changes in the mRNA levels of the cellular genes listed above, as well as *SERINC1* and *SERINC5*, were monitored over four days by RT-qPCR. SERINC5 caused no relevant changes in cellular mRNAs. In fact, their pattern of expression was comparable across treatments (Figure 7A–C). Consistent with their peak of expression, we observed a 2-fold increase in both *SERINC1* and *SERINC5* mRNAs 24 h and 48 h post-transfection (Figure 7D). Because these transfections were transient, *SERINC1* and *SERINC5* transcript levels started to wear off 96 h post-transfection. However, this decline was more prominent for *SERINC5* (>9-fold), which indicates that SERINC5 impacts its own expression (Figure 7D). These findings made us hypothesize that SERINC5 reduces the mRNA levels of genes that are not encoded in the cellular genome (ectopic or non-self-DNA). 

**Figure 8:**
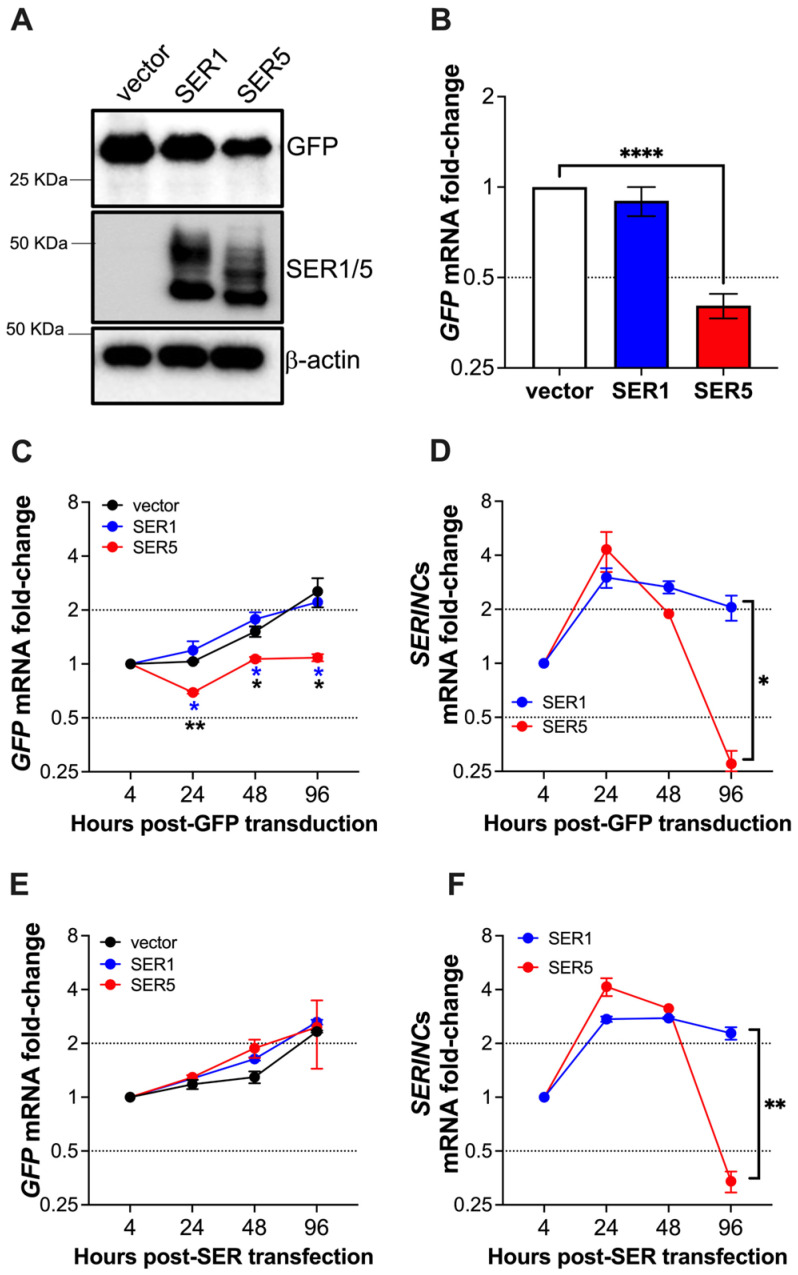
SERINC5 reduces mRNAs derived from non-self-DNA but has no impact on transcripts derived from self-DNA. HEK293T cells were co-transfected with a plasmid coding for *GFP* and either an empty vector, SERINC1-Myc-Flag, or SERINC5-HA constructs. Forty-eight hours later, the expression levels of *GFP* were measured by western blot (**A**) and RT-qPCR (**B**). (**C**,**D**) HEK293T cells were transfected with either an empty vector, SERINC1-Myc-Flag, or SERINC5-HA constructs. Three hours later, they were transduced with VLPs harboring *GFP*. The mRNA levels of *GFP* (**C**), *SERINC1*, and *SERINC5* (**D**) were examined by RT-qPCR for 96 h. (**E**,**F**) HEK293T cells stably expressing GFP were transfected with either an empty vector, SERINC1-Myc-Flag, or SERINC5-HA constructs. The mRNA levels of *GFP* (**E**), *SERINC1*, and *SERINC5* (**F**) were measured by RT-qPCR for 96 h. *: *p* < 0.05, **: *p* < 0.01, ****: *p* < 0.0001. Dotted lines represent the threshold for biological significance. Blots are representative of 3 independent assays. Data correspond to the mean and SEM of 3 independent experiments.

To test this, we transfected an expression vector coding for the Green Fluorescence Protein (GFP) in HEK293T cells, along with an empty vector, SERINC1-Myc-Flag, or SERINC5-HA. A reduction in GFP at the protein and mRNA levels was detected only in SERINC5-expressing cells (Figure 8A,B), confirming our hypothesis that SERINC5 attacks ectopic DNA. We then performed a similar assay, but in this case, *GFP* was encoded in a lentiviral genome instead of a plasmid, and we provided it to cells by transduction, which would mimic our HIV infection experiments. Cells were transfected with SERINC1/5 constructs and, 3 h later, they were transduced with lentiviral-like particles encoding *GFP*. *GFP* and *SERINC*s mRNAs were monitored for four days. Whereas *GFP* mRNAs steadily increased in the presence of the empty vector or SERINC1 until reaching their maximum expression at 96 h post-transduction, *GFP* expression was delayed in SERINC5^+^ cells, where transcripts never crossed the 2-fold threshold of expression (Figure 8C). Consistent with our findings in Figure 7, *SERINC5* mRNAs were significantly downregulated by 96 h post-transfection, whereas *SERINC1* transcripts were still 2-fold higher than on the day of transfection (Figure 8D). It is important to note that the timing and impact that SERINC5 has on its own expression versus *GFP* mRNAs are different. SERINC5 causes a significant delay in *GFP* transcription by 24 h post-transduction, while SERINC5‘s impact on its own expression starts to become noticeable 48 h post-transfection. These differences may be due to the experimental setup. Here, *GFP* is delivered by lentiviral transduction. Therefore, in order to produce *de novo* mRNA, the GFP construct needs to be reverse transcribed and integrated into the host genome, which is less efficient than if a vector coding for *GFP* was transfected to cells. Thus, in this scenario, the GFP construct is more vulnerable to SERINC5 restriction. Conversely, *SERINC5* is delivered to cells by transfecting a plasmid, so larger amounts of DNA are delivered compared to the transduction experiment (see the difference in magnitude of *GFP* mRNAs in vector or SERINC1 cells in Figure 8C compared to the *SERINC* mRNAs in Figure 8D). This may explain why it takes longer for SERINC5 to cause a significant impact on its own expression.

We next tested the effect of SERINC5 on cells that had been previously transduced with this GFP construct and were selected for its stable expression for over two weeks prior to the transfection with SERINC1 and SERINC5 plasmids. Here, no differences in *GFP* mRNA levels were observed in cells stably expressing GFP, regardless of the SERINC5 expression (Figure 8E). Once again, *SERINC5* was dramatically downregulated by 96 h post-transfection (Figure 8F). Therefore, these assays revealed that once retroviral integration has occurred, SERINC5 can no longer restrict gene expression, suggesting that SERINC5 is able to discriminate between self- and non-self-DNA.

### 3.5. SERINC5 Blocks Transcription of Non-Self-DNA

**Figure 9:**
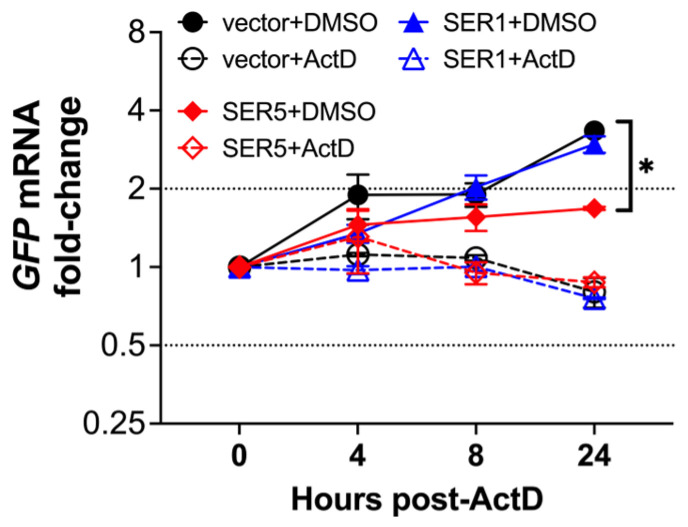
SERINC5 does not accelerate the turnover of non-self-RNA. HEK293T cells were co-transfected with a plasmid coding for *GFP* and either an empty vector, SERINC1-Myc-Flag, or SERINC5-HA constructs. Twenty-four hours later, 5 μg/mL of Actinomycin D (ActD) or DMSO were added. The RNA levels of *GFP* were examined 0, 4, 8, and 24 h after adding ActD by RT-qPCR. *: *p* < 0.05. Dotted lines represent the threshold for biological significance. Data correspond to the mean and SEM of 3 independent experiments.

The effect of SERINC5 on non-self-DNA expression can be caused by decreasing RNA stability, inhibiting transcription, promoting the degradation of ectopic DNA, or impacting the availability/transport of the ectopic DNA. To test whether SERINC5 impacts RNA stability, the mRNA levels of transfected *GFP* were evaluated over time in the presence and absence of a transcription inhibitor, Actinomycin D. The addition of Actinomycin D caused a similar reduction in *GFP* transcripts, regardless of the treatment (empty vector, SERINC1, or SERINC5) (Figure 9), indicating that SERINC5 does not accelerate RNA turnover. However, in the absence of Actinomycin D, the *GFP* mRNA levels were >2-fold lower in cells expressingSERINC5 (Figure 9). Thus, these findings reveal that SERINC5 downregulates the production of mRNA from non-self-DNA. 

**Figure 10:**
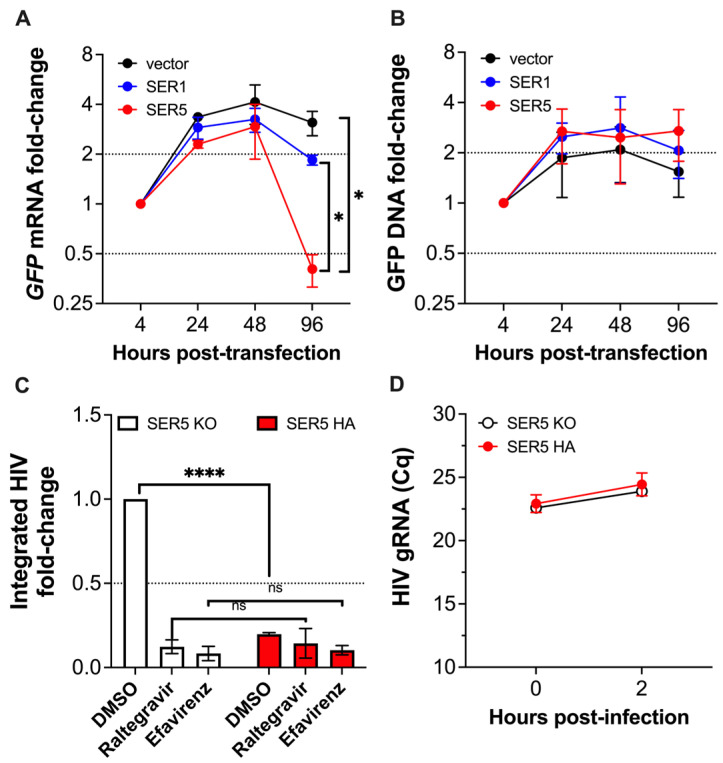
The SERINC5-dependent restriction of non-self-DNA is due to a defect in DNA availability. (**A**) Changes in the RNA and (**B**) DNA levels of the plasmid pCGCG-GFP were monitored over 4 days by qPCR in HEK293T cells co-transfected with an empty vector, SERINC1-Myc-Flag, and SERINC5-HA constructs. (**C**) Differences in HIV DNA integration for NL4-3Δ*nef* were investigated in JurkatTAg SERINC5-KO and parental JurkatTAg cells harboring an HA tag on the endogenous *SERINC5* gene. A reverse transcription inhibitor (Efavirenz, 40 μM) and an integration inhibitor (Raltegravir, 260 nM) were included as controls. Integrated HIV was measured by Alu-qPCR 16 h post-infection. (**D**) Differences in virus uptake between JurkatTAg SERINC5-KO and parental JurkatTAg were assessed by measuring HIV gRNA counts at 0 h and 2 h post-infection. Data are presented as raw Cq values. *: *p* < 0.05, ****: *p* < 0.0001. Dotted lines represent the threshold for biological significance. Data correspond to the mean and SEM of 3 independent experiments.

SERINC5 could downregulate mRNA from ectopic DNA by either promoting the degradation of the ectopic DNA template, impairing its transcription, or impacting DNA transport/availability. To test these hypotheses, we first monitored the mRNA and DNA levels of ectopic DNA (GFP, SERINC1, and SERINC5) over time by qPCR to assess whether SERINC5 causes DNA decay. Despite the dramatic downregulation of *GFP* transcripts in cells expressing SERINC5 (Figure 10A), no fluctuations in the GFP plasmid counts were detected (Figure 10B). To examine if SERINC5 affects DNA transport or availability, we used the JurkatTAg cell system. Since our data in Figure 8 suggests that the restriction of SERINC5 on retroviruses occurs at or before integration, we measured differences in HIV DNA integration between parental and SERINC5-KO cells infected with NL4-3Δ*nef*. As controls, infected cells were treated with reverse transcriptase and integrase inhibitors (Efavirenz and Raltegravir, respectively). As expected, only marginal integration was detected in cells treated with either of these compounds, regardless of SERINC5 expression. Remarkably, SERINC5 caused a 5-fold reduction in the amount of integrated HIV DNA, suggesting that SERINC5 impacts proviral DNA transport and/or integration efficiency (Figure 10C). To investigate if this effect is due to differences in virus uptake, we repeated the infection, and besides assessing HIV DNA integration, we also measured HIV gRNA by RT-qPCR right after spinoculation and 2 h later. A 2-Cq increase was detected 2 h post-infection compared to time zero for all experimental conditions, which reflects a decay in HIV gRNA (Figure 10D). This may in part be due to the fact that: (i) not all particles bound to cells successfully infect them, (ii) particles that successfully gain access to the cytoplasm start reverse transcription, which causes the degradation of the gRNA, and/or (iii) viral RNA is sensed and degraded by the host defenses. Regardless, the number of attached particles at time zero, as well as viral uptake at 2 h post-infection, is comparable between Parental and SERINC5-KO cells. Therefore, the defect in HIV DNA integration caused by SERINC5 is not due to differences in virus uptake. Overall, these results confirm that SERINC5 inhibits the transcription of non-self-DNA, likely by affecting its availability and/or integration efficiency, and that this is not due to DNA degradation.

### 3.6. SERINC5 Does Not Restrict SARS-CoV-2 RNA

**Figure 11:**
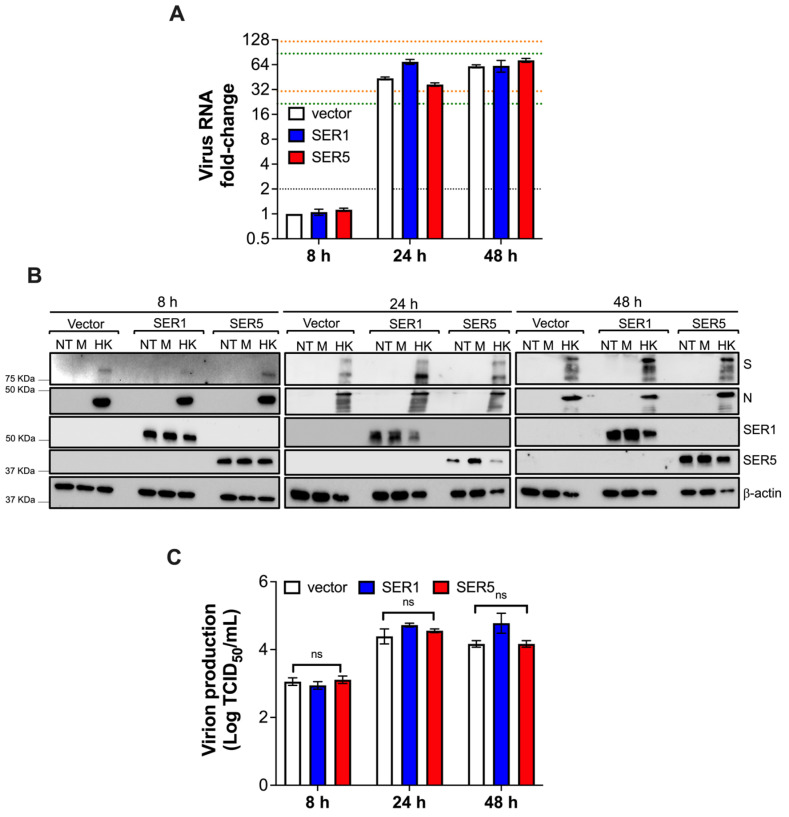
SERINC5 does not affect SARS-CoV-2 RNA. HEK293T-ACE2 cells stably expressing an empty vector, SERINC1-Myc-Flag, or SERINC5-HA were infected with the SARS-CoV-2 HK strain at MOI = 1. Next, 8, 24, and 48 h later, viral RNA levels, virus protein levels, and virion production were examined by RT-qPCR (**A**), western blot (**B**), and TCID_50_ (**C**), respectively. RNA levels were determined by RT-qPCR using primer pairs specific for the virus *RdRp* and *NSP6*. ns: not significant. Black dotted lines represent the threshold for biological significance for the 8 h time point. Green dotted lines represent the threshold for biological significance for the 24 h time point. Orange dotted lines represent the threshold for biological significance for the 48 h time point. Blots are representative of 3 independent assays. Data correspond to the mean and SEM of 3 independent experiments.

To investigate if besides ectopic DNA, SERINC5 similarly affects ectopic RNA, we infected ACE2^+^ and SERINC5-HA^+^ or SERINC1-Myc-Flag^+^ HEK293T cells with SARS-CoV-2 at MOI = 1. We included cells infected with lentiviral particles (mock infection) and non-infected (NT) cells as controls. Then, 8, 24, and 48 h post-infection, cells were harvested and analyzed for: (i) SARS-CoV-2 RNA levels (determined by RT-qPCR using primer pairs specific for the virus RNA-dependent RNA polymerase (RdRp) and NSP6), (ii) virus protein expression by western blot, and (iii) the production of infectious particles (measured by the median Tissue Culture Infectious Dose (TCID_50_) from the supernatants). While we observed some variations in the virus RNA levels in the presence of SERINC1 and SERINC5, these differences never reached the 2-fold threshold for biological significance [25] (Figure 11A, threshold represented by colored dotted lines). Consistent with this, no changes in the expression levels of the structural proteins S and N nor in virion production were detected over the two-day period (Figure 11B,C). A reduction in both SERINC1 and SERINC5 was detected 24 h and 48 h post-infection, but this was likely due to the cytopathic effects or the shutoff activity of the virus, since the actin levels were also considerably decreased (Figure 11B). Therefore, these findings indicate that SERINC5 does not impact virus RNA, at least not that of SARS-CoV-2.

## 4. Discussion

SERINC5 was identified in 2015 as an antiviral factor against retroviruses. Specifically, SERINC5 becomes incorporated into nascent virions and blocks their ability to infect new target cells. However, this effect is counteracted by HIV and SIV Nef [1,2]. Besides its impact on virus entry, two independent studies recently found that SERINC5 also inhibits virus gene expression and virion production in viruses unrelated to retroviruses, such as HBV and CSFV [21,22]. To evaluate if SERINC5 exerts similar antiviral actions on lentiviruses, we investigated the effect of SERINC5 on HIV and SIV protein levels and virion release. Similar to the CSFV study [22], we found that SERINC5 affects the expression of several lentiviral proteins, including Gag, which consequently reduces virion production. However, SERINC1, a protein with similar biological functions as SERINC5, does not reduce virus protein expression. Notably, as for its effect on virus entry, this activity of SERINC5 is counteracted by Nef, since the impact on virus protein levels and virion production is magnified in *nef*-deleted viruses, probably because SERINC5 was downregulated in Nef-expressing cells. However, it must be noted that these findings were observed under conditions of overexpression, which poses limitations to our study, since these effects could be observable only at high levels of SERINC5. To address this limitation, we examined this activity of SERINC5 in a more physiologically relevant system: using the endogenous levels of the protein and under conditions of infection. For this, we chose JurkatTAg cells. JurkatTAg cells deficient in SERINC5, as well as parental JurkatTAgs in which endogenous SERINC5 was tagged with HA, were infected with HIV-1 NL4-3 and NL4-3Δ*nef*. In line with our observations in transfected cells, virus proteins and virion production were impacted in SERINC5^+^ cells, and this effect was partly counteracted in the presence of Nef. To rule out if the effect of SERINC5 is specific against viruses or if it similarly affects host genes, we studied the effect of SERINC5 on cellular proteins that share a similar subcellular distribution to that of SERINC5: plasma membrane (IFNR1 and TNFR) and ER (Calreticulin). No changes in cellular gene expression were detected, even when fluctuations were investigated over a four-day period. Hence, these observations indicate that SERINC5 affects virus but not host protein expression, and because SERINC5 significantly downregulates Gag, virion production is also severely impacted. 

To uncover how SERINC5 reduces virus protein levels, we investigated whether SERINC5 promotes their proteasomal or lysosomal degradation using inhibitors for these pathways. Whereas an enhancement in SERINC5 expression was found under these conditions, which likely reflects the natural turnover of the protein through these two pathways, no restoration in virus protein levels was detected. On the contrary, due to the enhancement in SERINC5 expression, a further reduction in HIV proteins was observed. Since these assays exclude a role for SERINC5 in promoting protein degradation, we next examined if SERINC5 affects gene expression at the RNA level. Remarkably, we found a significant reduction in HIV RNAs, similarly affecting all splicing variants of the virus. Since the degree of SERINC5-mediated reduction of mRNAs is similar among HIV splicing forms (including un-spliced, single-spliced, and multiple-spliced transcripts) and *GFP* transcripts, which do not require splicing, we excluded splicing as a potential process impacted by SERINC5. A defect in RNA levels could be caused by a defect in transcription, but it could also be due to an impairment in the export of these transcripts to the cytosol. However, we also ruled this option out since HIV un-spliced, single-spliced, and multiple-spliced mRNAs, which use different mechanisms for RNA transport [37,38,39], were all similarly downregulated by SERINC5. While un-spliced and single-spliced HIV RNAs use the Rev-dependent export pathway, multiple-spliced RNAs and *GFP* transcripts use a Rev-independent pathway [38,39,40,41]. Notably, when we examined if the effect of SERINC5 could also be exerted on host mRNAs over time, we noticed that the mRNA levels for ectopically expressed *SERINC5* were impacted by its own expression. This made us hypothesize that SERINC5 targets non-self-DNA. This was corroborated by measuring fluctuations in ectopically expressed *GFP* in the presence and absence of SERINC5. To determine if SERINC5’s effect on RNA is specifically applied on non-self-DNA or if it could also be exercised upon non-self-RNA, we infected cells with SARS-CoV-2, a virus with a positive-sense, single-stranded RNA genome that contains signatures of mRNA [42,43,44,45]. Remarkably, SERINC5 did not cause major impacts on SARS-CoV-2 RNA, proteins, or infectious particle production, suggesting that either: (i) SARS-CoV-2 is equipped to counteract SERINC5, (ii) SERINC5 has no effects on ectopic RNA, or (iii) SERINC5 affects the synthesis of RNA that requires the cellular machinery. Recent studies have revealed that SARS-CoV-2 counteracts SERINC5 through two mechanisms. First, the virus uses ORF7a to prevent SERINC5 incorporation into nascent particles [19]. Second, SARS-CoV-2 generates small viral RNAs (svRNAs)—which work in an analogous manner to miRNAs—to decrease SERINC5 expression [46]. Hence, the lack of impact of SERINC5 on SARS-CoV-2 RNA and proteins may be due to these countermeasures. However, even when a virus can counteract a restriction factor, a partial impact on the step targeted by such factor is normally observed compared to cells that are deficient in its expression, and this would have become more evident here, particularly because SERINC5 is present in cells prior to SARS-CoV-2 infection and the expression of SERINC5 antagonists. Hence, the most likely explanation for our findings with SARS-CoV-2 is that either SERINC5 causes no defects in ectopic RNA, or that SERINC5 only impacts the synthesis of ectopic RNA that uses the cellular machinery (SARS-CoV-2 replicates in the cytosol using its own RNA-dependent RNA polymerase). Future experiments will test these two potential scenarios.

Since our results revealed that SERINC5 attacks non-self plasmid DNA, but our initial observations were made in the context of HIV/SIV (both transfection and infection), we investigated if SERINC5 could also attack proviral DNA that had not yet been integrated into the cell genome, which would be consistent with our observations for HIV/SIV transfections. For this, we examined differences in the mRNA levels of *GFP* that was delivered to cells by transduction or after transduction and selection of GFP-expressing cells. These two settings represent two different steps of a retroviral life cycle. In the first one, we assessed the overall impact of SERINC5 on a gene that needs to be reverse transcribed before integration and expression, while in the second scenario we studied if SERINC5 could affect the expression of that gene once it has been integrated into the human genome (and no longer considered non-self). Our assays uncovered that *GFP* expression was delayed after viral transduction in SERINC5-expressing cells, while in SERINC1-expressing cells the *GFP* transcripts tended to increase over time. In contrast, SERINC5 had no negative impact on the *GFP* mRNAs produced after proviral integration. Hence, these findings corroborate that SERINC5, or a SERINC5 effector, can discriminate between self- and non-self-DNA. 

To investigate how SERINC5 achieves the silencing of non-self-DNA, we explored the following hypotheses: (i) SERINC5 reduces DNA levels, (ii) SERINC5 increases RNA turnover, (iii) SERINC5 inhibits transcription, or (iv) SERINC5 reduces DNA availability. No significant changes in the DNA counts of a GFP plasmid were observed in SERINC5^+^ and SERINC5^−^ cells. Similarly, we did not find that SERINC5 accelerates RNA turnover of *GFP* transcripts, since in the presence of Actinomycin D, a transcriptional inhibitor [47], the fold-change of RNA yields was similar regardless of SERINC5 expression. We then reasoned that SERINC5 must affect transcription by either preventing the recruitment of the RNA Pol II and related transcription factors, by epigenetically silencing non-self-DNA, or by altering DNA transport/availability. To assess if SERINC5 alters DNA availability, we assessed differences in the amounts of HIV DNA integrated in the cellular genome by Alu-qPCR [26] in SERINC5-deficient and SERINC5-expressing cells. Remarkably, SERINC5 caused a 5-fold reduction in integrated DNA, which was not caused by a defect in virus infectivity or uptake. Therefore, SERINC5 impacts non-self-DNA availability and, in turn, its transcription.

However, several questions remain. How does SERINC5 affect the availability of non-self-DNA? Does SERINC5 work as a sensor by interacting with non-self-DNA? Does SERINC5 affect the nuclear import of ectopic DNA? Or is this effect part of the antiviral activity that SERINC5 triggers through its association with MDA5 [22]? We hypothesize that the actions of SERINC5 on non-self-DNA are indirect. While the role of SERINC5 in triggering an antiviral state through an interaction with MDA5 has been reported [22], and would be consistent with our observations here, our data suggest that the response is specific for DNA. In the study by Li and collaborators, the authors revealed that the SERINC5–MDA5 association triggers type I IFN signaling, even in the absence of an infection. Therefore, if cells become infected, this *milieu* favors an antiviral state in which thousands of ISGs are already induced. Some of these effectors are DNA sensors (i.e., cGAS), which would be expressed over basal levels, and thus, more prone to detect foreign DNA and silence it as a consequence of downstream effectors. However, one would argue that this antiviral environment would similarly affect expression from non-self-RNA, as the authors reported for CSFV [22]. In our study, we did not see such effect on SARS-CoV-2. Even when SARS-CoV-2 is equipped to counteract SERINC5 [19,46], we would expect a defect in replication since SERINC5 was already present in our cells prior to the infection and the subsequent synthesis of ORF7a and svRNAs—the SERINC5 antagonists in SARS-CoV-2. Thus, there must be a more specific mechanism causing the silencing of non-self-DNA, although MDA5 signaling might have an additive effect. 

Several lines of evidence point to SERINC5 localizing at the nuclear envelope [29,48], as well as the plasma membrane and ER. Our fluorescence microscopy assays have confirmed that SERINC5 can be found at perinuclear locations. This subcellular distribution is consistent with the MS proteomic study by Li and colleagues [22], in which several nuclear proteins appeared in the list of hits that physically interact with SERINC5. Among these hits, the authors found nucleosome assembly proteins, nuclear cap-binding proteins, Pol II subunit L, and TRIM28. We hypothesize that SERINC5 could impact the nuclear transport of these cellular factors or even non-self-DNA. In support of this hypothesis, a recent study has revealed that SERINC3/5 proteins cause defects in membrane asymmetry [49], which could be responsible for fusion defects at the virus entry step. Since SERINC5 is also present at the nuclear envelope, alterations of membrane asymmetry at the nuclear membrane could similarly affect nuclear transport. Alternatively, SERINC5 could induce a signaling cascade that predisposes the cell to silence ectopic DNA and/or might promote repressive epigenetic marks in non-self-DNA, which not only affects their expression but could also alter the integration efficiency of HIV proviral DNA into the host genome, consequently impacting HIV DNA availability [50]. Future work will test these hypotheses to uncover the molecular mechanism by which SERINC5 silences non-self-DNA. 

Remarkably, while we were finishing this work, another study reported that SERINC5 restricts HIV at a post-integration step, causing a significant reduction in HIV gene expression in myeloid cells [8]. While that study reported that SERINC5 on the surface of incoming particles is responsible for triggering this phenotype, here, we report that SERINC5 expressed in target cells can impact HIV transcription and that this block is exerted at a pre-integration step or during the integration step. Still, there may be commonalities between that report and the phenotype we are describing here, which reinforces the notion that we are only starting to understand SERINC5 actions in host defense.

## 5. Conclusions

Our findings demonstrated that SERINC5 is an important host defense protein. It not only causes a defect in virus infectivity, but also restricts the expression of non-self-DNA. Hence, the innate functions of SERINC5 extend beyond virus pathogens.

## Figures and Tables

**Table 1:** Antibody sources and conditions.

Protein Target	Antibody	Dilution	Source
β-actin	Mouse monoclonal (8H10D10) to β-actin	1:1000	Cell Signaling, #3700S
Calreticulin	Rabbit polyclonal to calreticulin	1:1000	Cell Signaling, #2891S
DAPI	N/A	1:5000	ThermoFisher Sci, #62248
GFP	Mouse monoclonal (GF28R) to GFP	1:1000	ThermoFisher Sci, #MA5-15256
HA	Mouse monoclonal (16B12) to HA tag	1:1000 WB 1:200 IF	BioLegend, #901502
HIV gp120	Goat polyclonal to HIV-1 gp120	1:1000	Abcam, #ab21179
HIV Nef	Mouse monoclonal to HIV-1 Nef	1:1000	ThermoFisher Sci, #MA1-71505
HIV p24 and SIV p27	Mouse monoclonal (183-H12-5C) to HIV-1 p24	1:1000	HIV Reagent Program, #ARP-3537
HIV Vif	Mouse monoclonal (319) to HIV-1 Vif	1:1000	Abcam, #ab66643
HIV Vpr	Rabbit polyclonal to HIV-1 Vpr	1:500	HIV Reagent Program, #ARP-11836
HIV Vpu	Rabbit polyclonal to HIV-1 Vpu	1:1000	HIV Reagent Program, #ARP-969
IFNR1	Rabbit monoclonal (EPR6244) to interferon α/β receptor 1	1:1000	Abcam, #ab124764
Myc	Mouse monoclonal (Myc.A7) to Myc tag	1:1000	Abcam, #ab18185
Myc	Rabbit polyclonal anti-Myc tag	1:200	Abcam, #ab9106
SARS-CoV-2 N	Rabbit monoclonal (8Q2E9) to SARS-CoV-2 Nucleoprotein	1:1000	ThermoFisher Sci, #MA5-36086
SARS-CoV-2 S	Rabbit polyclonal to SARS-CoV-2 Spike protein S1/S2	1:1000	ThermoFisher Sci, #PA5-112048
SIV Nef	Mouse monoclonal (17.2) to SIV Nef	1:1000	HIV Reagent Program, #ARP-2659
TNFR1	Rabbit monoclonal (C25C1) to TNFα receptor 1	1:1000	Cell Signaling, #3736S
Goat IgG	Donkey polyclonal (HRP) to goat IgG	1:6000	Abcam, #ab6885
Mouse IgG_1_	AlexaFluor 488 goat anti-mouse IgG_1_	1:500	ThermoFisher Sci, #A21121
Mouse IgG	Donkey polyclonal (HRP) to mouse IgG	1:4000	Pierce, #31430
Rabbit IgG	AlexaFluor 568 goat anti-rabbit IgG	1:500	ThermoFisher Sci, #A11011
Rabbit IgG	Goat polyclonal (HRP) to rabbit IgG	1:2500	Promega, #W4011
Rabbit IgG	Donkey polyclonal (HRP) to rabbit IgG	1:4000	Abcam, #ab16284

## Data Availability

Data are contained within this article or the Supplementary Materials.

## Supplementary Materials

The following supporting information can be downloaded at: https://www.mdpi.com/article/10.3390/v15091961/s1. Figure S1: SERINC1 and SERINC5 have a highly overlapped subcellular distribution. HeLa cells were co-transfected with SERINC1-Myc-Flag and SERINC5-HA constructs. Forty-eight hours later, cells were stained for HA (green), Myc (red), and the nucleus (DAPI, blue). Bar: 10 μm. Images are representative of 3 independent experiments. Figure S2: The SERINC5-mediated downregulation of lentiviral proteins is magnified in the absence of Nef. HEK293T cells were co-transfected with HIV-1 NL4-3 or NL4-3Δ*nef* and either an empty vector, SERINC1-Myc-Flag, or SERINC5-HA constructs. Forty-eight hours post-transfection, cells were harvested, and protein expression was measured by western blot. The levels of Gag (A), Vpr (B), Env (C), Vpu (D), and Nef (E) were measured by densitometry, normalized to β-actin, and expressed as relative expression compared to the vector control. *: *p* < 0.05, **: *p* < 0.01, ***: *p* < 0.001, ns: not significant. Data correspond to the mean and SEM of 3 independent experiments. Figure S3: Endogenous SERINC5 is degraded in proteasomes and lysosomes. JurkatTAg cells engineered to harbor an HA tag in the endogenous *SERINC5* gene were treated with DMSO, Hydroxychloroquine (Chlor; 20 μM), ALLN (5 μM), and MG132 (0.5 μM) for 18 h. Cells were then harvested and analyzed for HA-SERINC5 and β-actin by western blot. Blots are representative of 3 independent experiments. Figure S4: Schematic representation of the splicing process of the full-length HIV genomic RNA. Red arrows represent primer positions to detect single-spliced mRNA species. Green arrows represent primer positions to detect multiple-spliced mRNA species. Blue arrows represent primer positions to detect un-spliced HIV RNA.
