# Supplementary figures and images for: The Antiviral Factor SERINC5 Impairs the Expression of Non-Self-DNA
Source: Viruses. 2023 Sep 20;15(9):1961. doi: 10.3390/v15091961 (PMC10537789; doi:10.3390/v15091961)

**SER5-HA**

**SER1-Myc**

**DAPI**

**MERGE**

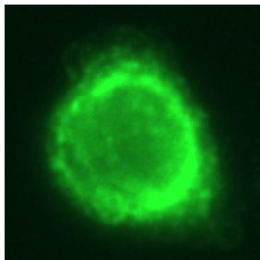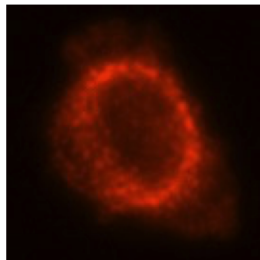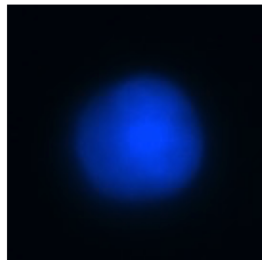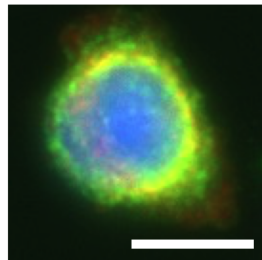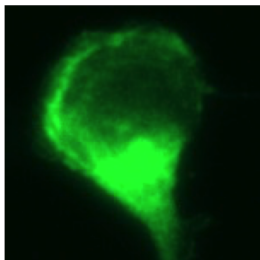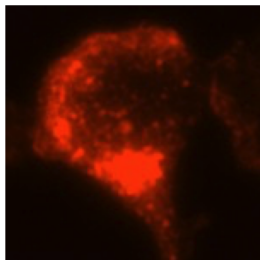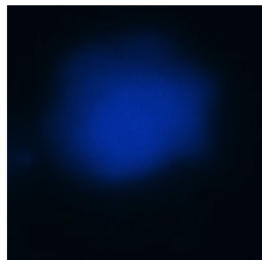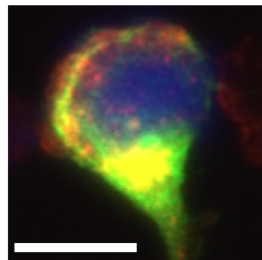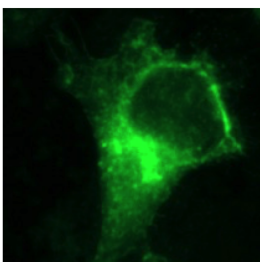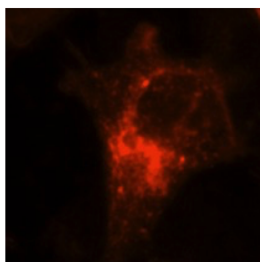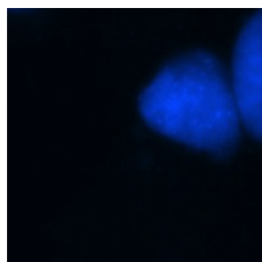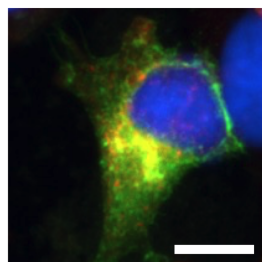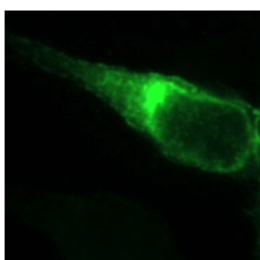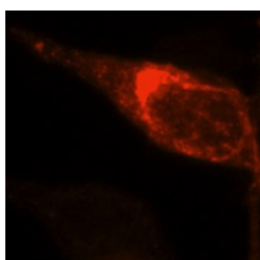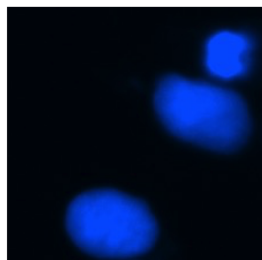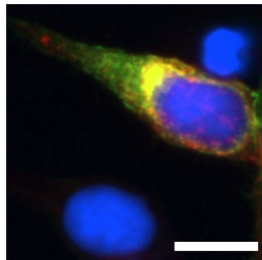

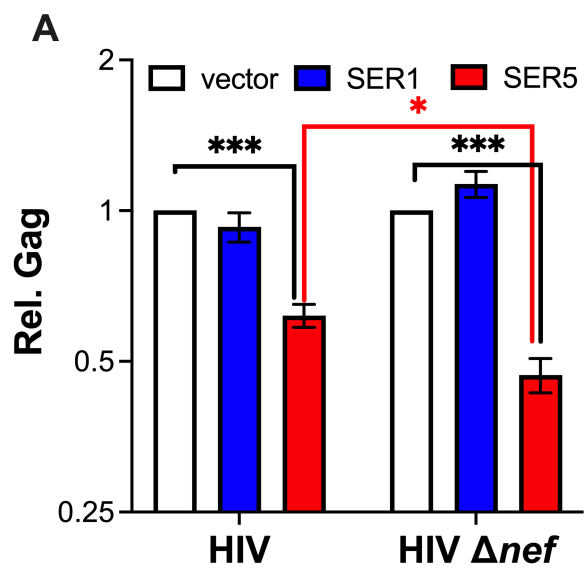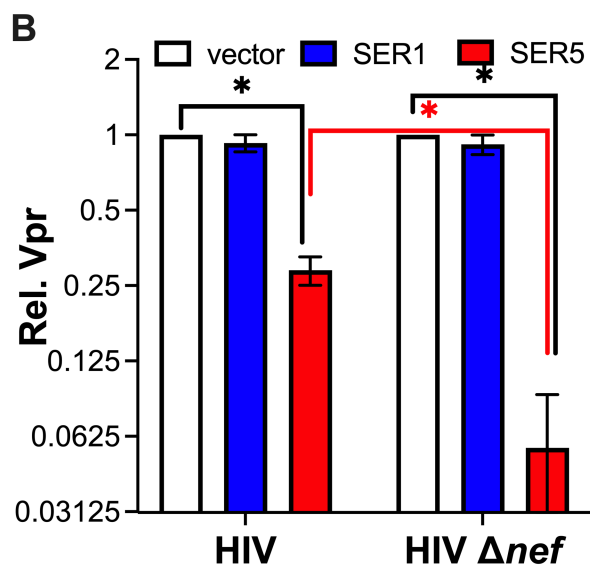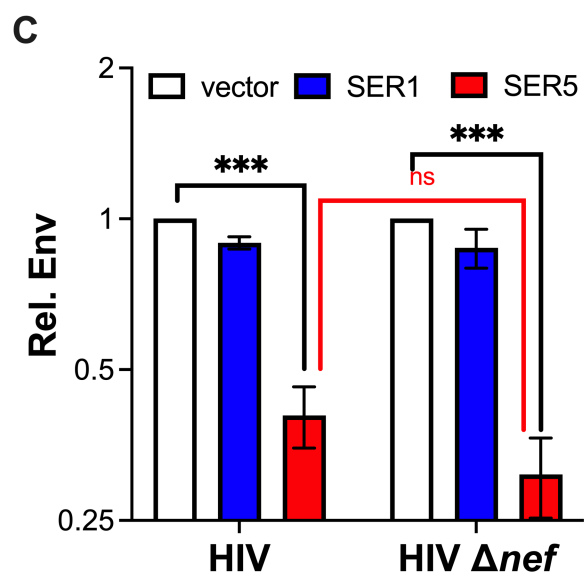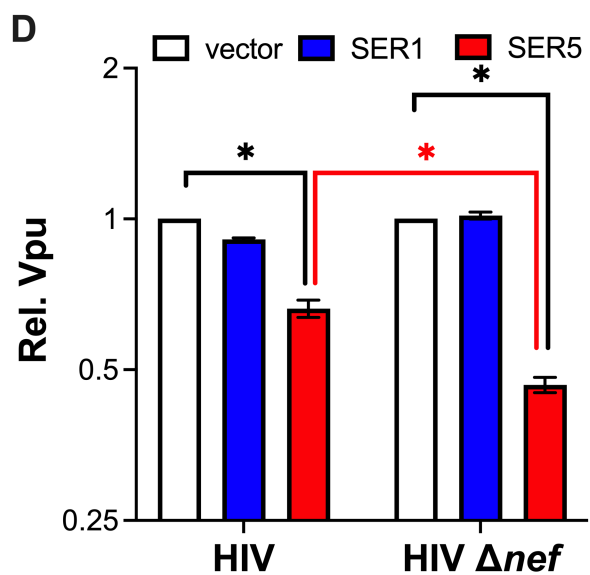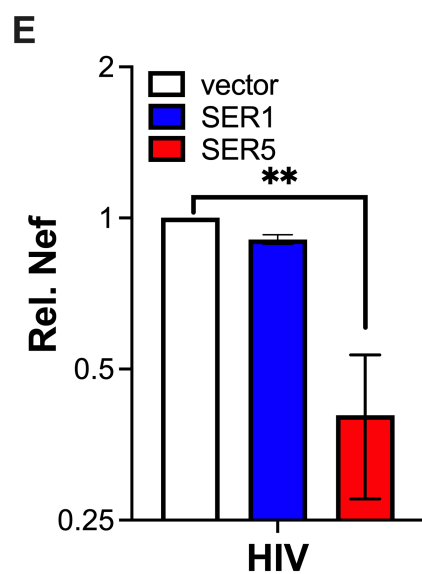

DMSO Chlor ALLN MG132

50 KDa —

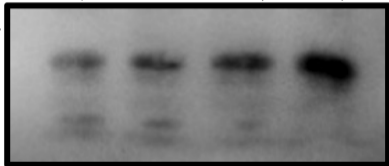

SER5

50 KDa —

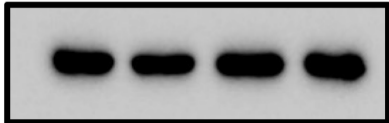

$\beta$ -actin

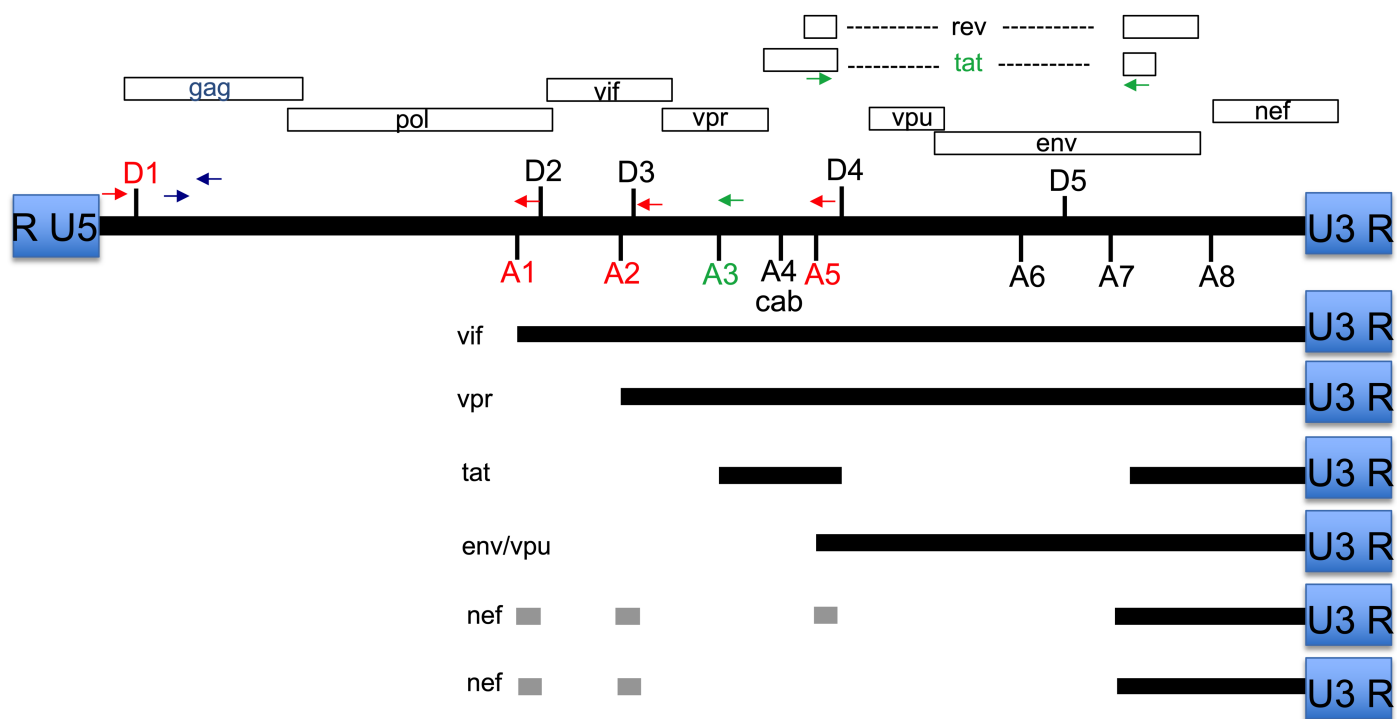

Supplement: Supplementary file 1 [file viruses-15-01961-s001.zip › viruses-2581310-supplementary.pdf]
